# Electro-Oxidation and Simultaneous Determination of Indole-3-Acetic Acid and Salicylic Acid on Graphene Hydrogel Modified Electrode

**DOI:** 10.3390/s19245483

**Published:** 2019-12-12

**Authors:** Xiaodong Cao, Xueting Zhu, Shudong He, Xuan Xu, Yongkang Ye

**Affiliations:** 1 School of Food and Biological Engineering, Hefei University of Technology, Hefei 230009, China; xiaodongcao@hfut.edu.cn (X.C.); xuetingzhu25@163.com (X.Z.); shudong.he@hfut.edu.cn (S.H.); 2 School of Chemistry and Molecular Engineering, Nanjing Tech University, Nanjing 211816, China; xuxuan0205@njtech.edu.cn

**Keywords:** electrochemical sensor, simultaneous detection, indole-3-acetic acid, salicylic acid, graphene hydrogel

## Abstract

A selective and sensitive electrochemical sensor was developed for simultaneous detection of phytohormones indole-3-acetic acid (IAA) and salicylic acid (SA). The sensing interface was fabricated on a porous, three-dimensional networked graphene hydrogel (GH) modified glassy carbon electrode (GCE). The electrocatalytic behavior of IAA and SA on the surface of the modified electrode (GH/GCE) was investigated by cyclic voltammetry and linear sweep voltammetry. Results show that the oxidation reactions of IAA and SA occur at different potentials, which enable their simultaneous detection at the sensing interface. Under optimal conditions, the GH/GCE exhibited good selectivity and stability and its response, unaffected by various interferents, was linear in the range of 4 to 200 μM of IAA and SA. The limit of detection (*S*/*N* = 3) achieved were 1.42 μM for IAA and 2.80 μM for SA. The sensor performance was validated by measuring for IAA and SA in real vegetable samples with satisfactory results.

## 1. Introduction

Plant hormones, also known as phytohormones, are organic chemicals produced in plants that can regulate physiological processes [1,2,3]. There are different classes of phytohormones, including abscisic acid, auxin, cytokinins, ethylene, gibberellins, and brassinosteroids. The concentrations of phytohormones present in plants are rather low and they vary during different growth periods. These phytohormones exert their effects in some complex and synergic ways and control all aspects of plant development—from embryogenesis, the regulation of organ size, pathogen defense, stress tolerance, and through to reproductive development [4]. Therefore, there is a need to develop simple and sensitive methods to detect and analyze phytohormones in plants [5]. Furthermore, there is an increasing interest now on analysis of multiple phytohormones, rather than a specific one [6].

The most powerful tools to simultaneously identify and determine multiple phytohormones are chromatographic techniques combined with sensitive detectors or tandem mass spectrometry [7,8,9,10,11]. Although such techniques possess advantages of excellent sensitivity and selectivity, efficient separation, and fast determination, they are limited to complicated and time-consuming sample preparation methods and require expensive apparatus to perform the test. Over the past decade, researchers have been pursuing alternative methods for the determination of phytohormones, including fluorescence spectrometry [1], enzyme-linked immunosorbent assay [12], and electrochemical analysis [13,14,15]. Among these, the electrochemical biosensors are the most promising given their inherent advantages of being simple, sensitive, rapid, portable, and cost-effective [11,13,14,16].

Indole-3-acetic acid (IAA) is the first identified auxin, which is regarded as the most important phytohormone [17]. IAA participates in the entire plant growth and development processes. It has been shown that IAA plays vital roles by interacting with other phytohormones under different conditions and asserting its effect on plant development [18]. Salicylic acid (SA), is also an indispensable phytohormone. SA plays a critical role in meditating the response of plants to abiotic stresses such as drought, temperature, heavy metal, and osmotic [19]. Some evidence shows that the crosstalk between SA and IAA signaling during the growth of plants is concentration dependent [19,20,21]. Therefore, it is necessary to develop simple, rapid, and sensitive methods to simultaneously detect and determine IAA and SA in plants.

In the last few years, several sensors have been fabricated on various nanomaterials to detect IAA and SA, especially using electrochemical methods [22,23]. A multiwalled carbon nanotubes-chitosan modified glassy carbon electrodes (GCE) was developed to detect both IAA and SA simultaneously in the range of 0.67 to 49 μM with a limit of detection (LOD) of 0.1 μM by using differential pulse voltammetry [23]. An electrochemical sensor, fabricated on a working electrode of carbon tape modified with carbon nanotubes, was used to monitor the amounts of IAA and SA during the development of pea roots [4].

Three-dimensional (3D) graphene materials which can be assembled by graphene nanosheets, have improved physical and electrochemical properties when compared to graphene nanosheets [24,25,26]. In recent years, they have gained great attention because of their high specific surface area, good electron mobility, porous, and 3D-networked structure [27,28,29]. Graphene hydrogel (GH) is a 3D graphene material that can be prepared easily by hydrothermal reduction [30,31,32] and chemical reduction [33,34]. To date, GH has been used in supercapacitors [30,34,35] and electrochemical sensors [36,37,38,39]. GH-based nanocomposites show excellent electrocatalytic activity to the oxidation of ascorbic acid (AA), dopamine (DA), and uric acid (UA), which has been used in their simultaneous detection in human serum samples [31,32]. In our previous work, we prepared gold nanoparticle-doped GH using hydrothermal method at temperatures higher than 100 °C, and its properties were investigated with various characterization methods. Furthermore, our work showed that it can be applied for amperometric determination of IAA and SA [36].

**Scheme 1:**
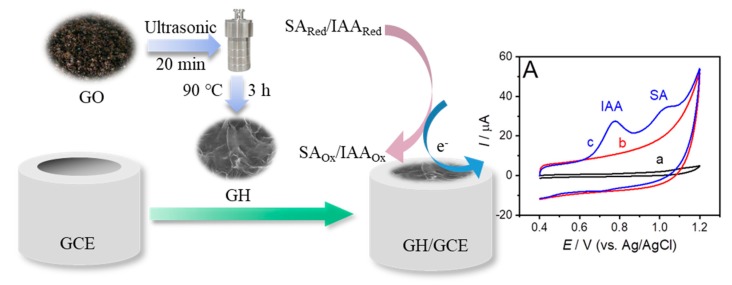
Preparation and detection procedures of the indole-3-acetic acid/salicylic acid (IAA/SA) sensor.

Herein, we prepared GH using the hydrothermal method at a relatively low temperature. Taking advantage of the porous and 3D-networked structure of GH, we constructed an electrochemical sensor for simultaneous detection of IAA and SA on a GH modified GCE (GH/GCE) (Scheme 1), and the electrooxidation behavior of IAA/SA were investigated as well. After the absorption on the GH/GCE, IAA and SA are readily oxidized within the potential window at different potentials facilitating their selective and simultaneous detection and quantification.

## 2. Experiment

### 2.1. Materials and Reagents

Graphite oxide was purchased from Nanjing Jicang Nano Technology Co., Ltd. (Nanjing, China). Nafion was bought from Sigma-Aldrich (St. Louis, MO, USA). IAA, SA, L-Cysteine (L-Cys), L-Tyrosine (L-Tyr), L-Tryptophane (L-Trp), L-Malic acid, citric acid (CA), abscisic acid (ABA), gibberellic acid (GA3), and sodium ascorbate were supplied by Aladdin (Shanghai, China). The 0.10 M stock solutions of IAA and SA were prepared using anhydrous ethanol and stored in a refrigerator. Phosphate buffer solution was prepared by mixing stock solutions of Na_2_HPO_4_ and NaH_2_PO_4_, and then adjusted to the desired pH by 0.10 M H_3_PO_4_. All other chemicals were of analytical grade and used as received. Deionized (DI) water was used all through the experiment.

### 2.2. Apparatus

Field emission scanning electron microscopy (SEM) images were obtained with SU8020 (Hitachi, Tokoyo, Japan) at an accelerate voltage of 5 kV. Raman characterization was measured on a LabRam HR Evolution (HORIBA Jobin Yvon, Paris, France) at the *λ*_exc_ of 532 nm.

Electrochemical characterizations and measurements were performed on a CHI 660D electrochemical workstation (Shanghai Chenhua, Shanghai, China) with a standard three-electrode configuration. A platinum wire provided the counter electrode, an Ag/AgCl electrode acted as the reference, and the 3 mm diameter GH modified GCE (GH/GCE) was used as the working electrode. Electrochemical impedance spectroscopy (EIS) measurements were conducted in 0.10 M KCl containing 5 mM (Fe(CN))^3−/4−^ with the frequency ranging from 0.1 Hz to 10 kHz. The electrochemical behaviors of IAA and SA were investigated by cyclic voltammetry (CV) in a 5.0 mL phosphate buffer (0.10 M, pH 2.5) at the scan rate of 100 mV s^−1^, either in the potential window 1.20 to −0.40 V with the initial potential of −0.40 V, or in 1.20 to 0.40 V window with the initial potential of 0.40 V. Linear sweep voltammetry (LSV) measurements for simultaneous determination of IAA and SA were performed in a 5 mL phosphate buffer spiked with various known concentrations of IAA (4–200 μM) and SA (4–200 μM) at the scan rate of 100 mV s^−1^ within a potential window of 0.40 to 1.20 V. An absorption procedure of IAA and SA onto the surface of the GH/GCE was performed by dipping the electrode in the above phosphate buffer solution dispersions containing various concentrations of IAA and SA under stirring for 150 s before conducting CV and LSV measurements. Chronoamperometry was used to investigate interference of other substances of IAA and SA. The amperometric responses of possible interfering substances to IAA and SA were measured at the optimized potentials of 0.80 and 1.00 V, respectively.

### 2.3. Procedures

#### 2.3.1. Synthesis of GH

Graphene oxide (GO) suspension was obtained by ultrasonication of graphite oxide (3.0 mg mL^−1^) according to the published method [40,41]. The suspension was then dried for later use. The GH was prepared according to Xie et al. with minor modification [33]. Typically, 10.0 mL of GO suspension (1.20 mg mL^−1^) was sonicated for 20 min, and then 66.0 mg of sodium ascorbate was added under ultrasonication. The pH of the above dispersion was adjusted by using NaOH or H_2_SO_4_ solution to 3.5, 5.3, 7.4 and 9.5, respectively. The mixture was sealed in an autoclave and heated at 90 °C for 3 h to form the GHs. The GH obtained were freeze-dried for 24 h to form the GH powder. The as-prepared GHs were respectively denoted as GH-3.5, GH-5.3, GH-7.4 and GH-9.5, where the numbers represent the solution pH during preparation.

#### 2.3.2. Preparation of GH Modified GCE

The GCE was polished with 0.10 and 0.05 μM alumina slurry and sequentially cleaned by sonication with acetone, ethanol, and water. The GH powder (1.0 mg) was dispersed in 2.0 mL Nafion (0.2%) solution by ultrasonication to form a GH solution of 0.5 mg mL^−1^. Then, 5.0 µL of the GH solution was dropped onto the surface of GCE. The GH/GCE were dried at room temperature. The GO/GCE and bare GCE were also prepared as control electrodes.

### 2.4. Sample Analysis

Celery and young tomato plant leaves were acquired locally in Hefei, China. They were cut and ground into a fine powder in the presence of liquid nitrogen. Then, 2.0 mL of pre-chilled methanol (4 °C, 80%) was added into the powdered sample (0.5 g) under shaking at 4 °C overnight, after which 1.0 mL of CHCl_3_ was added and the mixture was shaken for another 4 min. Finally, the mixture was centrifuged at 12,000 rpm for 5 min under 4 °C. The supernatant was collected and dried at room temperature under nitrogen. The detection samples were prepared by dissolving the dried extractions with anhydrous ethanol and then diluting with a phosphate buffer solution (0.1 M, pH 2.5) to a final volume of 5.0 mL.

## 3. Results and Discussion

### 3.1. Characterization of GH

**Figure 1:**
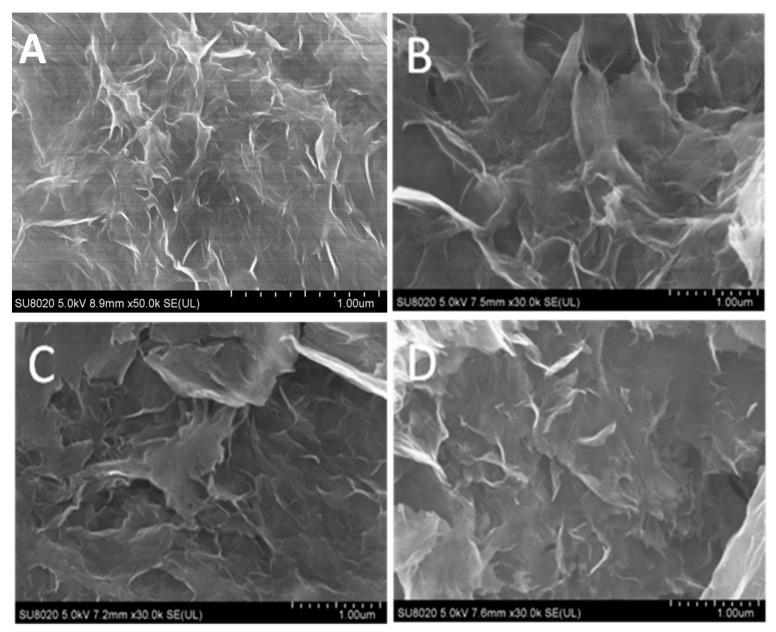
SEM images of (**A**) graphene hydrogel (GH)-3.5, (**B**) GH-5.3, (**C**) GH-7.4, and (**D**) GH-9.5.

The morphology of the GHs prepared under different pH values were characterized by SEM (Figure 1). All GHs showed porous 3D-networked structure, though the pH value at which they were prepared affected the internal structure significantly. The GH-3.5 (Figure 1A) and GH-5.3 (Figure 1B) samples exhibited dense and fluffy structure, and the pore walls consisted layers of stacked graphene unbroken nanosheets. In GH-7.4 (Figure 1C) and GH-9.5 (Figure 1D) samples, the porous structure was uncompacted, and the pore walls were cracked. These indicate that the GHs prepared at low pHs had better mechanical property than those prepared at high pHs [33], which we attribute to the high density of the networked structure and stacking of graphene nanosheets.

**Figure 2:**
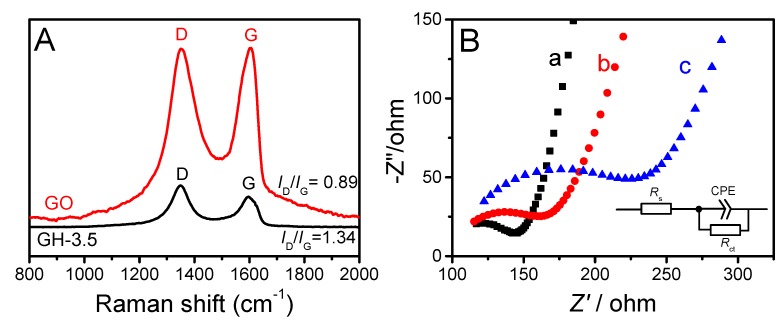
(**A**) Typical Raman spectra of GO and GH-3.5. (**B**) Nyquist plots of bare GCE (a), GH-3.5/GCE (b), and GO/GCE (c), inset shows the equivalent circuit used for fitting the impedance data.

Figure 2A shows the typical Raman spectra of GO and GH-3.5, which displays two characteristic bands at about 1350 (D band) and 1600 cm^−1^ (G band). The D band corresponds to the A_1g_ breathing mode of the six-atom ring (sp^3^ bonded carbons), and the G band relates to the high frequency E_2g_ phonon of the sp^2^ bonded carbons [42,43]. According to the Raman spectra, the I_D_/I_G_ ratio in GH is 1.34, while that of GO is 0.86. This indicates a decrease in the average size of sp^2^ sites in GH and alteration in the structure of GO during the reduction process with increasing structural defects [44,45]. The GH-3.5 was also characterized by EIS. As can be seen in Figure 2B, the inset is a fitting simulation circuit of the Nyquist spectrum of EIS, the fitting data are listed in Table S1 (Support Information). Here, the constant phase element (CPE) is defined by two values, CPE-T and CPE-P. CPE-P can indicate how close it is to a standard capacitor. If CPE-P equals 1 then the CPE is identical to that of a capacitor. The solution resistance *R*_s_ values obtained at three electrodes are quite close. The values of the electron transfer resistance *R*_ct_ are 82.8 ± 5.1 Ω (bare GCE), 119.2 ± 3.7 Ω (GH-3.5/GCE), and 169.6 ± 5.6 Ω (GO/GCE). This indicates that GH-3.5 is more conductive than GO, which makes it a potential nanomaterial in sensor fabrication.

### 3.2. Electrochemical Oxidation of IAA and SA on GH/GCE

**Figure 3:**
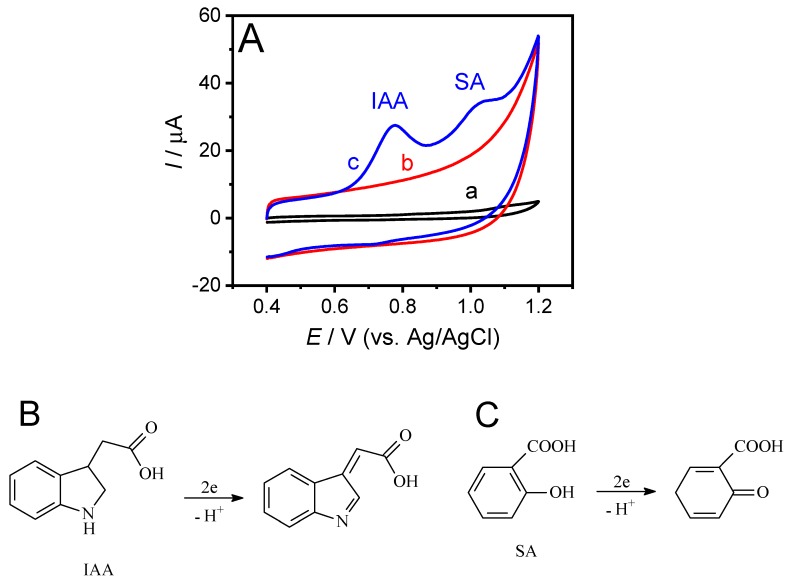
(**A**) Cyclic voltammetry curves (CVs) of bare glassy carbon electrode (GCE) and GH-3.5/GCE in the absence of IAA and SA in 0.1 M pH 2.5 phosphate buffer solution, and CV of GH-3.5/GCE in the presence of 40 µM IAA and 20 µM SA in 0.1 M pH 2.5 phosphate buffer solution Scan rate: 100 mV s^−1^. (**B**) Possible electrochemical oxidation reactions of IAA and (**C**) SA.

**Figure 4:**
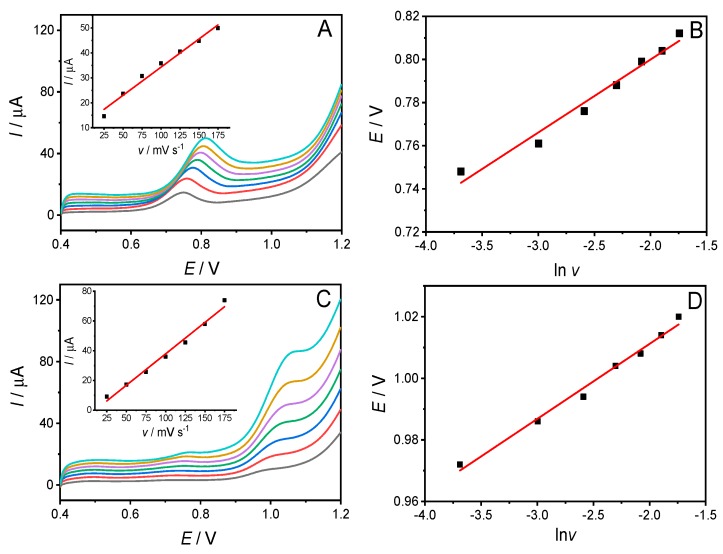
(**A**) CVs of IAA at GH/GCE with scan rate (*v*) from 25 to 175 mV s^−1^ (from bottom to top). Inset: The linear relationship between *i*_pa_ and *v*. (**B**) The relationship between *E*_pa_ of IAA and ln*v*. (**C**) CVs of SA at GH/GCE with *v* from 25 to 175 mV s^−1^ (from bottom to top). Inset: The linear relationship between *i*_pa_ and *v*. (**D**) The relationship between *E*_pa_ of SA and ln*v*.

**Figure 5:**
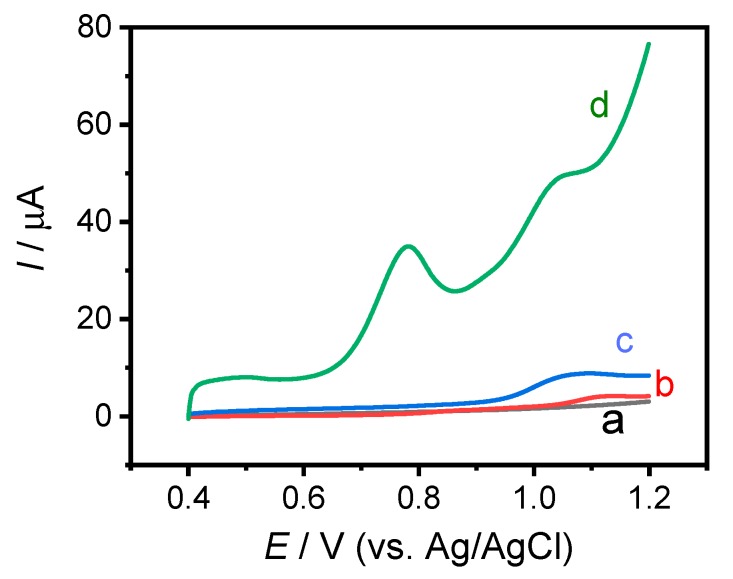
Linear sweep voltammetry curves (LSVs) recorded on different electrodes in 0.1 M pH 2.5 phosphate buffer solution at scan rate of 100 mV s^−1^. (**a**,**b**) bare GCE in the absence and presence of IAA (80 µM) and SA (80 µM), respectively; (**c**) GO/CCE in the presence of IAA (80 µM) and SA (80 µM); and (**d**) GH-3.5/GCE the presence of IAA (80 µM) and SA (80 µM). Scan rate: 100 mV s^−1^.

The electrochemical behaviors of 40.0 µM IAA and 20.0 µM SA in a 0.10 M pH 2.5 phosphate buffer solution at GH/GCE were investigated by CV in a potential range from 0.4 to 1.2 V (Figure 3A). No redox peaks were observed on bare and GH modified GCE in 0.10 M pH 2.5 phosphate buffer solution without IAA and SA. However, after absorption of IAA and SA for 150 s, there were two obvious peaks at about 0.78 and 1.00 V at GH/GCE in buffer solution containing 40.0 µM IAA and 20.0 µM SA. Based on the literature, the peak at 0.78 V was assigned to IAA and the peak at 1.00 V to SA [23,24]. The oxidation peak currents of IAA (Figure 4A) and SA (Figure 4C) increased with the increase of *v* from 25 to 175 mV s^−1^, and the *E*_pa_ shifted positively with the increasing scan rate. The relationships between *i*_pa_ of IAA (Figure 4A, inset) and SA (Figure 4C, inset) were linear with scan rate. These indicate that the irreversible electrode reactions of IAA and SA were adsorption controlled [23]. Meanwhile, the oxidation peak potentials (*E*_pa_) of IAA/SA were linearly with ln*v* (Figure 4B,D), which were in accordance with Laviron’s Equation [46]:

$$E_{p}=E^{0}+\frac{RT}{\alpha nF}\ln\left(\frac{RTk^{0}}{\alpha nF}\right)-\frac{RT}{\alpha nF}\ln v$$

where *α* is the transfer coefficient, *k*^0^ is the apparent rate constant of the surface reaction, *v* is the scan rate and *E*^0^ is the formal potential. Assuming *α* = 0.5, the electrons involved in the oxidation of IAA and SA were calculated to be ≈2. Moreover, the electrochemical oxidation reaction of IAA/SA on GH/GCE was also affected by the pH value of phosphate buffer solution (Figure S1, Supporting Information). The oxidation peak of IAA/SA shifted negatively with the increasing pH, indicating a proton involved reaction. The irreversible reaction of IAA/SA on the surface of GH/GCE may participate as two electrons and one proton [23], which can be expressed as follows (Figure 3B,C):

Afterword, the improved response of IAA/SA at GH/GCE was investigated by LSV measurement. Figure 5 illustrates the electrocatalytic ability of bare GCE, GO/GCE, and GH-3.5/GCE to IAA (80 µM) and SA (80 µM) by the LSV measurement within a potential window from 0.4 to 1.2 V. No oxidation peak observed on bare GCE in the absence of IAA and SA, which agreed with the result of CV measurement (Figure 5, curve a). Broad oxidation peaks appeared on the bare GCE (Figure 5, curve b) and GO (Figure 5, curve c) modified GCE in the presence of IAA and SA. The peaks observed on bare GCE and GO/GCE were located at about 1.13 and 1.09 V, respectively. However, two obvious and separate oxidation peaks at about 0.78 and 1.00 V were present at GH-3.5/GCE (Figure 5, curve d). The negative shifts of these oxidation peaks indicate that IAA and SA can be oxidized easier on GH modified GCE than on bare and GO modified GCE. The improved electrocatalytic catalytic ability of GH to IAA and SA may due to the increasing defect of GH synthesized from chemical reduction of GO. Furthermore, the peak currents at GH-3.5/GCE were much higher than those at GO/GCE and bare GCE. This indicates that GH/GCE is much more sensitive to IAA and SA than the other two electrodes.

### 3.3. Electrochemical Detection of IAA and SA

As can be also observed in Figure S1, the oxidation peak current of IAA/SA decreased sharply with increasing pH from 2.5 to 4.5. Moreover, the oxidation peak of SA almost disappeared when the pH value of phosphate buffer solution increased to 4.5. The pH value of GH preparation showed an effect on the analytical performance of the chemical sensor as well. The GH-3.5 gave the best electrocatalytic performance towards IAA/SA in the detecting buffer with a pH value of 2.5 (Figure S2, Supporting Information). Thus, GH-3.5/GCE and the detecting buffer of pH 2.5 were used in the detection of IAA and SA afterward.

**Figure 6:**
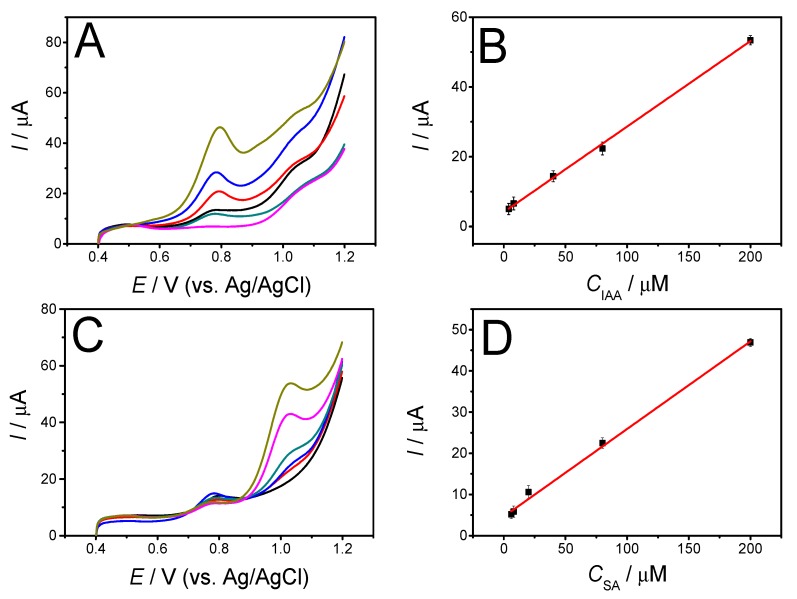
(**A**) LSVs on GH/GCE in 0.1 M pH 2.5 phosphate buffer solution containing different concentrations of IAA (from 4 to 200 μM). The LSVs were obtained in the presence of 6 μM SA at the scan rate of 100 mV s^−1^. (**B**) Calibration curve between anodic peak current of IAA and the concentration of IAA. (**C**) LSVs on GH/GCE in 0.1 M pH 2.5 phosphate buffer solution containing different concentrations of SA (from 6 to 300 μM). LSVs were obtained in the presence of 6 μM IAA at the scan rate of 100 mV s^−1^. (**D**) Calibration curve between anodic peak current of SA and the concentration of SA.

The individual detection of IAA and SA on GH/GCE were carried out by LSV measurement. As shown in Figure 6, using the IAA and SA mixture when increasing the concentration of one and keeping the other unchanged, the oxidation peak current increased linearly with changing constituent, unaffected by the unchanged constituent. For example, in the presence of 6.0 μM SA, IAA can be detected from 4.0 to 200.0 μM (Figure 6A,B), and in the presence of 6.0 μM IAA, SA can be detected from 6.0 to 300.0 μM in phosphate buffer solution (Figure 6C,D), with LODs (*S*/*N* = 3) of 1.43 μM for IAA, and 1.79 μM for SA. The linear relationship between the sensor current (*I*, μA) and target concentration (*C*, μM) are as follows:

For IAA: *I*_IAA_ = 0.25*C*_IAA_ + 4.21 (*R*^2^ = 0.999)

For SA: *I*_SA_ = 0.22*C*_SA_ + 4.65 (*R*^2^ = 0.997)

**Figure 7:**
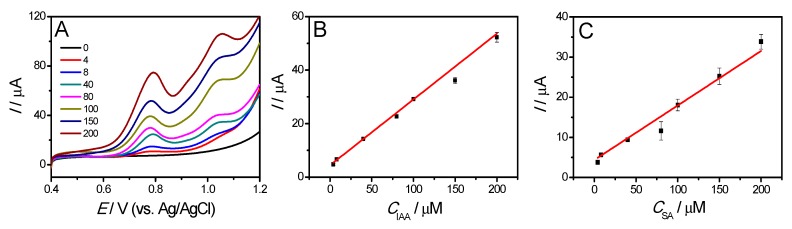
(**A**) LSVs on GH/GCE in 0.1 M pH 2.5 phosphate buffer solution containing various concentrations of IAA and SA. Scan rate: 100 mV s^−1^. (**B**) Calibration curve between anodic peak current of IAA and the concentration of IAA. (**C**) Calibration curve between anodic peak current of SA and the concentration of SA.

The results of simultaneous determination of both IAA and SA are shown in Figure 7. The oxidation peak currents at different potentials of IAA and SA increased simultaneously with increasing concentrations, with LODs (*S*/*N* = 3) of 1.42 μM for IAA and 2.80 μM for SA. The linear calibration curves of IAA and SA in the tested concentration range from 4.0 to 200.0 μM are as follows:

For IAA: *I*_IAA_ = 0.25 *C*_IAA_ + 4.43 (*R*^2^ = 0.997)

For SA: *I*_SA_ = 0.14 *C*_SA_ + 4.36 (*R*^2^ = 0.972)

The performance characteristics of our sensor, i.e., linear range and LOD, are comparable to those of other electrochemical sensors for both simultaneous and individual detection of IAA and SA (Table 1). Our sensor also exhibited good analytical performance in detecting IAA/SA.

### 3.4. The Selectivity, Reproducibility, and Stability of the GH/GCE

Amperometric measurements were performed to determine the selectivity of GH/GCE towards IAA/SA in the presence of interferents such as CA, ABA, GA3, AA, L-malic acid, L-Trp, L-Tyr, and L-Cys. Before measurements, the working potentials were first optimized in 0.1 M phosphate buffer solution with a pH value of 2.5, and the results are displayed in Figure S3. As can be seen from the figure, the optimal working potential for amperometric measurements of IAA and SA were 0.8 V (Figure S2A, Supporting Information) and 1.0 V (Figure S3B, Supporting Information), respectively. The amperometric responses to IAA/SA and other substances are exhibited in Figure S3. The results (Figure S3, Supporting Information) show that the GH/GCE was very robust and its performance for IAA was unaffected by the presence of those interferents. However, the presence of 20.0 μM IAA interfered with SA measurement when using amperometric measurement. This may be due to the amperometric response of IAA on GH/GCE at 1.0 V (Figure S3A, Supporting Information). Nonetheless, the above results indicate that the GH/GCE has good selectivity to IAA and SA.

Three GH/GCEs were fabricated to evaluate the fabrication reproducibility of the sensor. These GH/GCEs were used to simultaneous detection of 80.0 μM IAA and 60.0 μM SA in 0.10 M pH 2.5 phosphate buffer solution using LSV. The relative standard deviations (RSDs) of IAA and SA were 7.2% and 9.5% (*n* = 3), respectively. The reproducibility of our sensor was assessed by detecting IAA (80.0 μM) and SA (60.0 μM) five times in succession with corresponding RSDs of 2.5% and 3.5%. The storage (at 4 °C) stability of GH/GCE was also investigated. The results show that the LSV response currents of IAA (80.0 μM) and SA (60.0 μM) decreased respectively by 2.1% and 4.0%, after five days and by 4.3% and 9.1%, after 10 days. These results indicate the acceptable reproducibility and good stability of our sensor.

### 3.5. Detection of IAA and SA in Real Samples

The proposed method was finally applied for the determination of IAA and SA in celery and tomato leaf samples, and the accuracy of the method was verified by recovery experiments. Using the calibration data (Figure 7B,C), the amount of IAA and SA present in celery and tomato leaf samples were determined as 5.02 and 4.00 μM, and 3.98 and 4.12 μM, respectively. Further to determine the recoveries, we spiked real samples with 4.0 and 40.0 μM each of IAA and SA. The recovery results (spiked plus initial) were in the range from 94.9% to 105.2% (Table 2). These results indicate excellent sensor performance.

## 4. Conclusions

The electrochemical oxidation of IAA/SA were investigated, and a selective and sensitive electrochemical sensor based on GH modified GCE was developed for simultaneous determination of IAA and SA. The prepared GH exhibited a porous and 3D networked structure, good conductivity, and excellent electrocatalytic activity, which enabled the GH/GCE to realize sensitive determination of IAA and SA. As IAA and SA were electrochemically oxidized at different potentials on the GH/GCE, and the oxidation currents of IAA and SA were proportional to the concentration of the detecting substances, the proposed sensor shows its ability for selective and simultaneous determination of IAA and SA. The developed method was demonstrated to have excellent accuracy in detecting IAA and SA in real vegetable samples, which makes it a potential sensor for practical detection of IAA and SA in plants.

## Figures and Tables

**Table 1:** Performance of different electrochemical sensors for the detection of IAA and/or SA.

Electrode *	Substance	Potential/V	Method	Linear Range/μM	LOD/μM	Ref.
CMC-MMT-SWCNT/GCE	IAA SA	0.89 (vs. SCE) 1.18 (vs. SCE)	LSV	0.005–0.30, 0.30–70.0 (with 80 μM SA) 0.01–300 (with 40 μM IAA)	0.002 0.0063	[23]
MWCNT-CS/GCE	IAA SA	0.72 (vs. SCE) 0.88 (vs. SCE)	DPV	0.67–48.82 0.67–48.82	0.10 0.10	[22]
BDD	IAA	0.93 (vs. Ag/AgCl)	SWV	5–50	1.22	[47]
SPE	SA	--	SWV	16–300	5.60	[48]
CNT-PABS/MAGNP/PADC/ITO	SA	1.14 (vs. Ag/AgCl)	CV	6–100	0.105	[49]
Au@Fe_3_O_4_/GCE	SA	--	DPV	1.0–1200.0	0.10	[50]
CFE	SA	--	DPV	2.0–3000.0	1.68	[51]
PNP/Pt	SA	--	i-t	20–500	6.40	[52]
GH/GCE	IAA SA	0.78 (vs. Ag/AgCl) 1.00 (vs. Ag/AgCl)	LSV	4–200 4–200	1.42 2.80	This work

* Carboxymethyl cellulose (CMC), montmorillonite (MMT), and single-walled carbon nanotube (SWCNT) modified GCE; multiwalled carbon nanotubes (MWCNT)-chitosan (CS) modified GCE; boron-doped diamond electrode (BDD); carbon paste electrode (SPE); anionic carbon nanotubes (CNT-PABS), cationic iron oxide nanoparticles (MAGNP), and cationic poly(diallyl dimethylammonium) hydrochloride (PDAC) modified indium doped tin oxide (ITO) substrates; Au@Fe_3_O_4_ and CS modified GCE; carbon-fiber electrode (CFE); Platinum nanoparticles (PNP) modified platinum disk electrode (Pt).

**Table 2:** Results of the recovery analysis of IAA and SA in vegetable samples (*n* = 3).

Sample	IAA Added (μM)	SA Added (μM)	IAA Detected (μM)	SA Detected (μM)	Recovery of IAA (%)	Recovery of SA (%)
Celery	--	--	5.02	3.98		
	40.00	40.00	45.30 ± 0.07	42.50 ± 0.23	100.6 ± 0.16	96.6 ± 0.52
Tomato leaves	--	--	4.00	4.12		
	4.00	4.00	7.59 ± 0.04	8.54 ± 0.11	94.9 ± 0.50	105.2 ± 1.35

## Supplementary Materials

The following are available online at https://www.mdpi.com/1424-8220/19/24/5483/s1, Figure S1. LSV recorded on GH-3.5 modified GCE at 100 mV s−1 in the presence of 80 μM IAA and 60 μM SA in PBS at different pHs. Figure S2. LSV of GH/GCE of 100 mV s−1 in the presence of 80 μM IAA and 60 μM SA in 0.10 M PBS at pH 2.5 using GCEs modified with different GHs. Figure S3. Working potential optimization for amperometric measurements of IAA (10 μM) (**A**) and SA (20 μM) (**B**). Figure S4. (**A**) Amperometric response of GH/GCE in 0.10 M PBS (pH 2.5) at +0.80 V for the addition of 10 μM IAA; 200 μM CA, ABA, GA3, and L-Malic acid; 20 μM L-Tyr, SA, AA L-Trp, and L-Cys. (**B**) Amperometric response of GH/GCE in 0.10 M PBS (pH 2.5) at +1.00 V for the addition of 10 μM SA; 200 μM CA, ABA, GA3, and L-Malic acid; 20 μM L-Tyr, AA, L-Trp, L-Cys, and IAA. Table S1. Fitting values of the equivalent circuit elements for different working electrodes (WE).
