# Supplementary material for: Electro-Oxidation and Simultaneous Determination of Indole-3-Acetic Acid and Salicylic Acid on Graphene Hydrogel Modified Electrode
Source: Sensors (Basel). 2019 Dec 12;19(24):5483. doi: 10.3390/s19245483 (PMC6960803; doi:10.3390/s19245483)
Supplement: Supplementary file 1 [file sensors-19-05483-s001.pdf]

sensors

Electronic Supporting Information

Electro-Oxidation and Simultaneous Determination of Indole-3-Acetic Acid and Salicylic Acid on Graphene Hydrogel Modified Electrode

Influence of pH

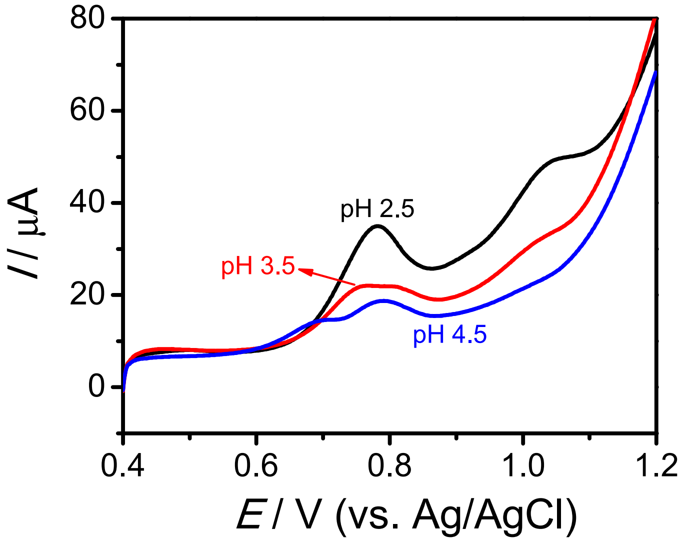

Figure S1. LSV recorded on GH-3.5 modified GCE at 100 mV s^{-1} in the presence of 80 μM IAA and 60 μM SA in PBS at different pHs.

Parameter Optimization

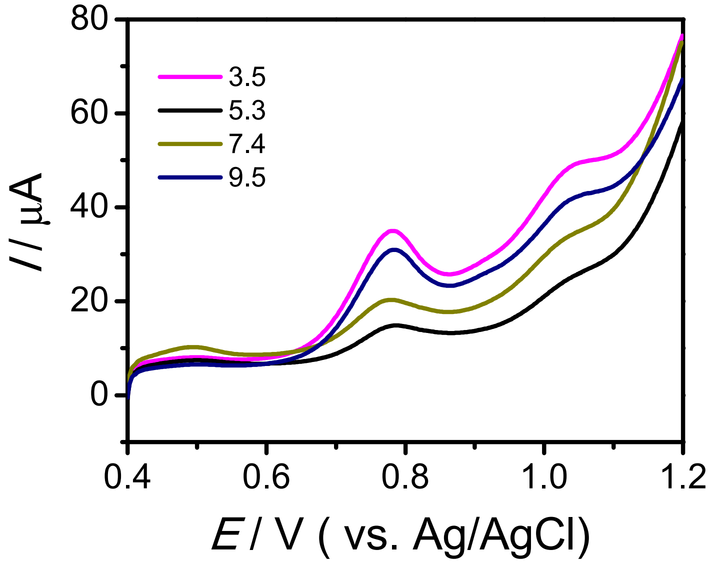

Figure S2. LSV of GH/GCE of 100 mV s^{-1} in the presence of 80 μM IAA and 60 μM SA in 0.10 M PBS at pH 2.5 using GCEs modified with different GHs.

Working Potential Optimization

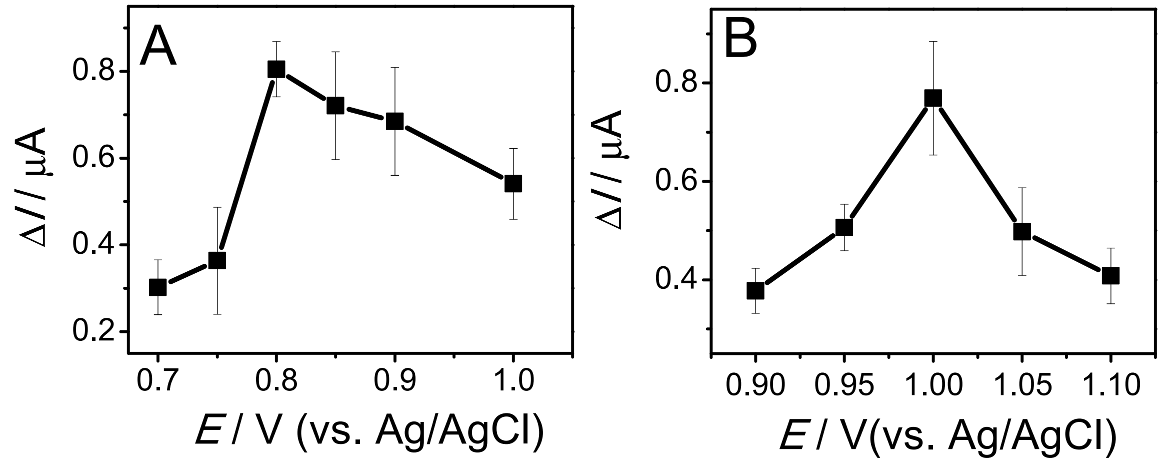

Figure S3. Working potential optimization for amperometric measurements of IAA (10 μM) (**A**) and SA (20 μM) (**B**).

The Selectivity of GH/GCE to IAA and SA

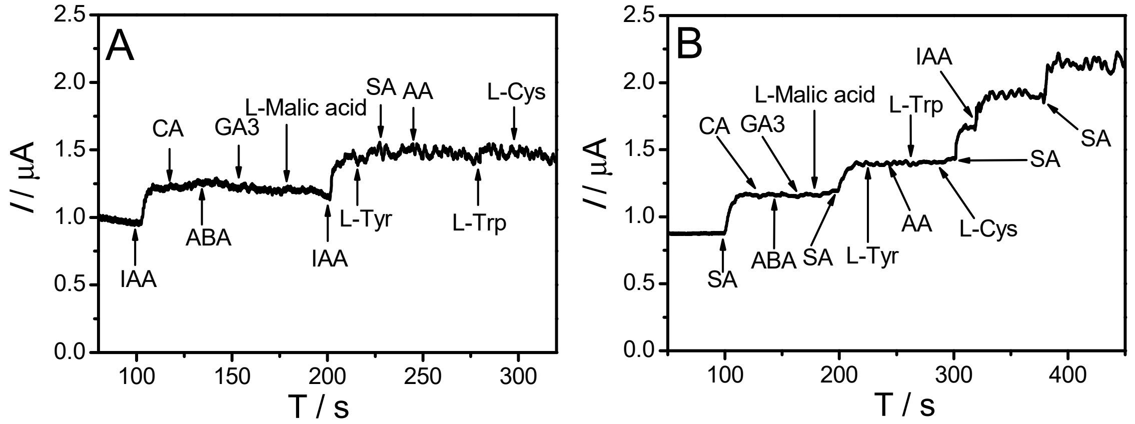

Figure S4. (**A**) Amperometric response of GH/GCE in 0.10 M PBS (pH 2.5) at +0.80 V for the addition of 10 μM IAA; 200 μM CA, ABA, GA3, and L-Malic acid; 20 μM L-Tyr, SA, AA L-Trp, and L-Cys. (**B**) Amperometric response of GH/GCE in 0.10 M PBS (pH 2.5) at +1.00 V for the addition of 10 μM SA; 200 μM CA, ABA, GA3, and L-Malic acid; 20 μM L-Tyr, AA, L-Trp, L-Cys, and IAA.

Table S1. Fitting values of the equivalent circuit elements for different working electrodes (WE).

WE	R_s (Ω)	CPE-T (μF cm^{-2})	CPE-P	R_{ct} (Ω)
**Bare GCE**	79.6 ± 2.79	13.9 ± 2.43	0.63 ± 0.02	76.9 ± 3.70
**GO/GCE**	95.1 ± 1.34	6.4 ± 0.95	0.74 ± 0.02	169.4 ± 5.57
**GH/GCE**	91.6 ± 0.88	6.1 ± 0.57	0.73 ± 0.01	108.3 ± 1.95

© 2019 by the authors. Licensee MDPI, Basel, Switzerland. This article is an open access article distributed under the terms and conditions of the Creative Commons Attribution (CC BY) license (http://creativecommons.org/licenses/by/4.0/).
